# Mavacamten in the Treatment of Obstructive Hypertrophic Cardiomyopathy: from Pathophysiology To Real World Data

**DOI:** 10.1007/s11886-025-02316-6

**Published:** 2025-12-01

**Authors:** Giorgia Panichella, Manuel Garofalo, Maddalena Ragagnin, Angela Ilaria Fanizzi, Mattia Zampieri, Annamaria Del Franco, Francesco Cappelli, Maurizio Pieroni, Iacopo Olivotto

**Affiliations:** 1 Department of Experimental and Clinical Medicine, University of Florence, Florence, Italy; 2 Cardiomyopathy Unit, Careggi University Hospital, Largo Brambilla, 3 , Florence , 50134 Firenze Italy; 3 Pediatric Cardiology, Meyer Children’s Hospital, Florence, Italy

**Keywords:** Cardiac myosin inhibitors, LVOT obstruction, Reverse remodeling, Genotype-guided therapy, Symptom burden, Cost-effectiveness

## Abstract

**Purpose of Review:**

This review aims to provide a comprehensive overview of mavacamten in the management of obstructive hypertrophic cardiomyopathy (oHCM), from its molecular mechanism of action to clinical trial outcomes and real-world application. It explores the efficacy, safety, and practical considerations of mavacamten use, while highlighting evolving indications and future directions.

**Recent Findings:**

Randomized trials have shown that mavacamten significantly reduces left ventricular outflow tract gradients, improves symptoms and exercise capacity, and induces structural reverse remodeling with a favorable safety profile. Real-world data confirm these benefits in broader patient populations. Pharmacogenetic variability, titration protocols, and cost-effectiveness analyses have further refined its clinical use.

**Summary:**

Mavacamten represents a paradigm shift in oHCM treatment by targeting disease at its sarcomeric origin. Its integration into routine care is expanding, supported by real-world evidence. Ongoing studies will clarify its role in non-obstructive HCM, heart failure with preserved ejection fraction, pediatric populations, and early-stage disease.

## Introduction

Hypertrophic obstructive cardiomyopathy (oHCM) is a common inherited disorder characterized by myocardial hypercontractility and impaired relaxation, leading to progressive left ventricular (LV) hypertrophy and dynamic left ventricular outflow tract (LVOT) obstruction. Traditional therapy includes non-specific negative inotropes or septal reduction therapies (SRTs), but these do not address the disease’s molecular basis [1].

The development of cardiac myosin inhibitors (CMIs) represents a new era in the treatment of oHCM [2]. These agents directly modulate the contractile apparatus of the cardiomyocyte by attenuating the hypercontractile phenotype at its molecular origin. Mavacamten, a first-in-class allosteric inhibitor of cardiac myosin adenosine triphosphatase (ATPase), has shown promising results in both preclinical and clinical settings, demonstrating significant reductions in LVOT gradients and improvements in symptoms, functional capacity, and quality of life [3, 4].

This review aims to provide a comprehensive overview of mavacamten in the treatment of oHCM. We will first explore the molecular basis of mavacamten mechanism of action, followed by efficacy and safety data from randomized clinical trials (RCTs). Finally, we will examine the emerging body of real-world evidence, which offers valuable insights into the drug’s performance outside controlled study settings.

## Mechanism of Action of Mavacamten

Cardiac myosin is the main sarcomeric motor protein and is responsible for force generation through its interaction with actin. It is composed of two heavy chains, each with a globular head domain with ATPase activity [2]. In HCM, pathogenic variants in sarcomeric proteins - most commonly *MYBPC3* (myosin-binding protein C) and *MYH7* (myosin-7) - disrupt the normal sarcomeric contractility and energy utilization [5]. These variants induce a pathological shift in the conformational equilibrium of β-cardiac myosin away from the super-relaxed state (SRX) - a highly autoinhibited conformation with markedly reduced ATPase activity - toward the disordered relaxed state (DRX), in which one of the two myosin heads becomes available for ATP hydrolysis and actin binding [6–9]. Under physiological conditions, this SRX/DRX balance ensures that only ~ 10% of myosin heads participate in contraction at any given time [10]. However, sarcomeric variants destabilize SRX head-head interactions, expanding the pool of myosin heads engaged in cross-bridge cycling [11–13]. This hypercontractility precedes the development of overt hypertrophy, diastolic dysfunction, and increased myocardial energy demand [14].

**Fig. 1:**
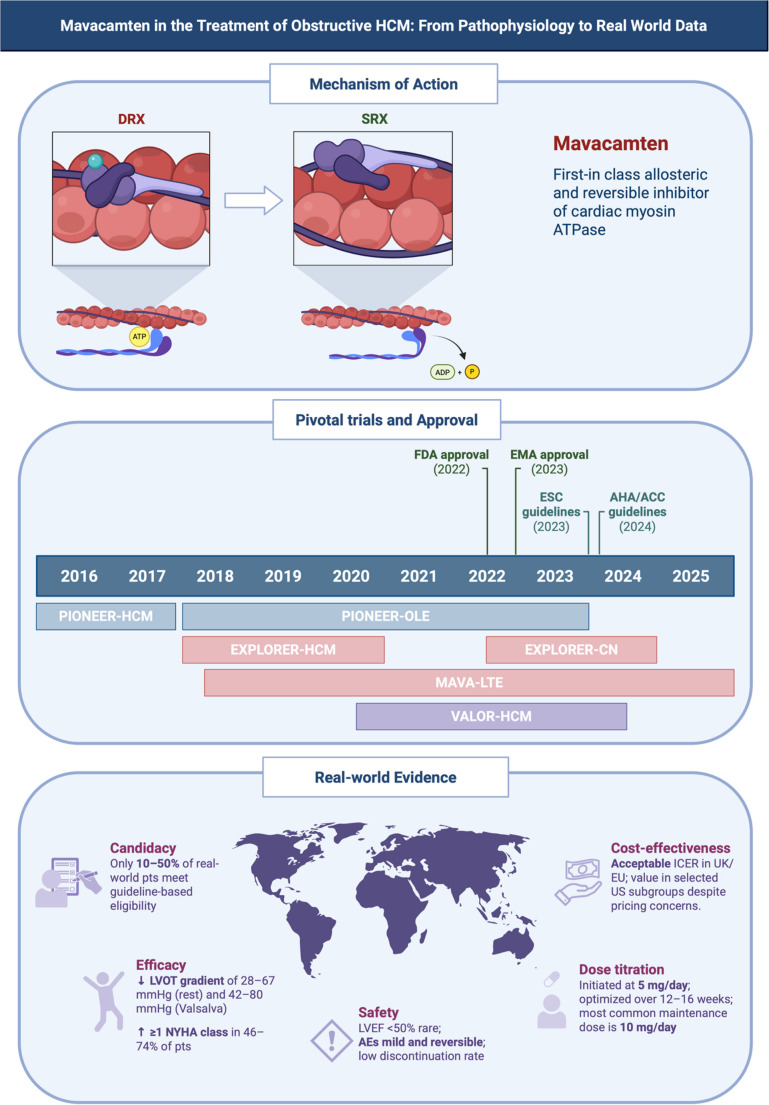
Mavacamten in the treatment of obstructive hypertrophic cardiomyopathy (oHCM): from pathophysiology to real-world data. **Top panel**. Mechanism of action of mavacamten showing its role in shifting myosin heads from the active disordered relaxed (DRX) state to the energy-conserving super-relaxed (SRX) state, reducing excessive ATPase activity and actin-myosin cross-bridging. **Middle panel.** Timeline of pivotal trials (PIONEER, EXPLORER, VALOR-HCM) and key milestones including FDA and EMA approval, as well as incorporation into ESC and ACC/AHA guidelines. **Bottom panel.** Summary of real-world evidence across five key domains: candidacy, efficacy, safety, dose titration, and cost-effectiveness. ACC, American College of Cardiology; AEs, adverse events; AHA, American Heart Association; DRX, disordered relaxed state; EMA, European Medicines Agency; ESC, European Society of Cardiology; FDA, U.S. Food and Drug Administration; ICER, incremental cost-effectiveness ratio; LVEF, left ventricular ejection fraction; LVOT, left ventricular outflow tract; NYHA, New York Heart Association; oHCM, obstructive hypertrophic cardiomyopathy; pts, patients; REMS, Risk Evaluation and Mitigation Strategy; SRX, super-relaxed state. (Created in BioRender. Cappelli, F. (2025) License link: https://BioRender.com/x19u79w.)

Mavacamten (previously MYK-461) is a first-in-class allosteric and reversible inhibitor of cardiac myosin ATPase [2]. It binds to β-cardiac myosin and reduces its enzymatic activity by stabilizing the SRX conformation and limiting the number of myosin heads available to engage with actin [2] (Fig. 1). It reduces actin-activated ATPase activity in a dose-dependent manner, primarily by slowing the release of inorganic phosphate during the actomyosin chemo-mechanical cycle - a key rate-limiting step of force production [15, 16]. Crucially, mavacamten reverses the abnormal SRX/DRX ratio observed in HCM-causing variants [5, 7].

Altered myofilament Ca²⁺ sensitivity is a key contributor to hypercontractility and diastolic dysfunction in HCM, as it lowers the threshold for actin–myosin interaction. This is often accompanied by impaired Ca²⁺ reuptake and abnormal cytosolic handling [17]. Mavacamten has been shown to reduce both maximal force and Ca²⁺ sensitivity, while preserving length-dependent activation, thus maintaining Frank–Starling responsiveness [18–20]. Moreover, in cellular models of thin filament HCM variants, the drug partially normalized Ca²⁺ sensitivity and reduced pathological elevations in peak systolic Ca²⁺ [21].

Beyond its effects on sarcomeric contractility, mavacamten also exhibits disease-modifying properties when administered in early-stage HCM. In pre-hypertrophic transgenic mice harboring pathogenic *MYH7* variants, chronic mavacamten treatment not only reduced contractility but also prevented the onset of myocardial hypertrophy, cellular disarray, and interstitial fibrosis [15]. Histological analysis after 20–26 weeks of therapy revealed up to an 80% reduction in fibrotic burden compared to placebo, while transcriptomic profiling showed normalization of hypertrophic and profibrotic gene expression signatures [15]. Notably, these protective effects were not observed when treatment was initiated after hypertrophy had already developed, underscoring the importance of early intervention.

In summary, mavacamten acts directly at the level of the sarcomere to normalize the pathologically increased availability of force-generating myosin heads seen in HCM. By stabilizing the SRX state and reducing ATPase activity, it favors an energy-sparing state that impacts the molecular progression of disease [22].

## Clinical Trials Evidence

### Pivotal Trials

First clinical evidence emerged from PIONEER-HCM (NCT02842242), an open-label phase 2 study that enrolled 21 symptomatic patients with oHCM and preserved left ventricular ejection fraction (LVEF ≥ 55%) [23]. Mavacamten significantly reduced the post-exercise LVOT gradient and improved peak oxygen consumption (pVO₂), New York Heart Association (NYHA) class, and quality of life scores, with dose-dependent effects [23]. The follow-up study PIONEER-OLE (Open Label Extension, NCT03496168) confirmed the effectiveness and safety of long-term mavacamten therapy in 13 patients up to 260 weeks [24] (Table 1).

**Table 1:** Overview of pivotal clinical trials evaluating Mavacamten in obstructive hypertrophic cardiomyopathy

Trial	Study Design	Number of patients	Duration	Inclusion Criteria	Drugs and Dose Tested	Primary endopoint	Secondary endpoint	Key Results
Phase II								
PIONEER-HCM (NCT02842242)	Non-randomized open-label pivotal clinical trial	*N* = 21	12 weeks (completed)	oHCM, baseline LVEF ≥ 55%, NYHA class II/III	Cohort A (*n* = 11) 10–20 mg of mavacamten with no background medications Cohort B (*n* = 10) 2–5 mg mavacamten with β-blockers allowed	Change in post-exercise peak LVOT gradient	Changes in pVO_2_, resting and Valsalva LVOT gradients, LVEF, numerical rating scale dyspnea score, NYHA class, NT-proBNP, KCCQ score	Mavacamten reduced mean postexercise LVOT gradient and increased peak pVO_2_ and symptoms in both cohorts of patients with oHCM. The effect was more pronounced in the cohort without background therapy. Mavacamten was well tolerated, with mostly mild (80%) adverse effects.
PIONEER-OLE (NCT03496168)	Non-randomized open-label extension clinical trial	*N* = 13	Up to 260 weeks (completed)	Completion of PIONEER-HCM	Starting dose 5 mg and following dose titration 5–15 mg of mavacamten with β-blockers and calcium-channel blockers allowed	Safety evaluation: frequency and severity of adverse events and serious adverse events. Efficacy evaluation using individualized dosing.	-	Demonstration of continued safety and effectiveness of mavacamten for > 3 years in oHCM. At week 180, mavacamten was associated with NYHA class improvement, sustained reductions in LVOT gradients and serum NT-proBNP levels and improved KCCQ-OSS.
Phase III								
EXPLORER-HCM (NCT03470545)	Randomized double-blind placebo-controlled (1:1 mavacamten-placebo) clinical trial	*N* = 251	30 weeks (completed)	oHCM, baseline LVEF ≥ 55%, NYHA class II/III, able to perform upright CPET, SpO2 ≥ 90% at rest	2.5–15 mg mavacamten with β-blockers and calcium-channel blockers allowed compared to placebo	Positive clinical response defined as: - pVO_2_ ≥ 1.5 mL/kg/ min + NYHA class reduction ≥ 1 OR - pVO_2_ ≥ 3.0 mL/kg/min without NYHA class reduction	Peak post-exercise LVOT gradient, pVO2, NYHA class reduction ≥ 1, Health related quality-of-life by KCCQ score, severity of HCM symptoms assessed by HCMSQ score	A complete clinical response was achieved in 27.4% of treated patients, compared with 0.8% in the placebo group. Mavacamten improves exercise capacity, LVOT obstruction, NYHA class, and health status.
EXPLORER-CN (NCT05174416)	Randomized double-blind placebo-controlled (2:1 mavacamten-placebo) clinical trial conducted in China with a long-term extension	*N* = 81	30 and 78 weeks (active)	oHCM, baseline LVEF ≥ 55%, NYHA class II/III, non-pregnant and non-lactating	1–15 mg mavacamten vs. placebo with β-blockers and calcium-channel blockers allowed	Change in peak Valsalva LVOT gradient	Peak resting LVOT gradient, proportion of patients achieving Valsalva LVOT peak gradient < 30mmHg and < 50mmHg, NYHA class reduction ≥ 1, KCCQ-CCS, change from baseline in NT-proBNP, cardiac troponin or left ventricular mass index	Mavacamten effective and safe in Chinese patients. 48.1% of -treated patiens achieved a Valsalva LVOT peak gradient < 30 mmHg compared to 3.7% of the placebo group. Mavacamten led to significant improvements also in secondary endpoints, the safety was similar between mavacameten and placebo.
VALOR-HCM (NCT04349072)	Randomized double-blind, placebo- controlled clinical trial	*N* = 112	16 and 56 weeks (completed)	oHCM with recommendation for invasive therapies, referred or under active consideration for SRT within past 12 months, baseline LVEF ≥ 60%, SpO2 ≥ 90% at rest	2.5–15 mg mavacamten vs. placebo with β-blockers, calcium-channel blockers and disopyramide allowed	Composite of decision to proceed with SRT and SRT-guideline eligible	NYHA class reduction ≥ 1, KCCQ-CSS, change from baseline in NT-proBNP, in cardiac troponin or in peak post-exercise LVOT gradient	In oHCM patients with intractable symptoms, mavacamten significantly reduced the fraction of patients meeting guideline criteria for SRT after 16 weeks of treatment (17.9% of mavacamten treated-patients vs. 76.8% of the placebo-group). The cross-over study confirmed the efficacy of mavacamten at 128 weeks.
MAVA-LTE (NCT03723655)	Randomized, double-blind, parallel, phase II/III clinical trial	*N* = 282	Up to 252 weeks (active)	Completion of Parent Study (MAVERICK-HCM or EXPLORER-HCM), oHCM for EXPLORER Cohort, nHCM for MAVERICK Cohort, baseline LVEF ≥ 50%, non-pregnant, non-lactating	Explorer Cohort: 2.5–15 mg mavacamten with β-blockers and calcium-channel blockers allowed Maverick Cohort: 2.5–15 mg with target mavacamten plasma concentrations of either 200 or 500 ng/mL	Safety evaluation: frequency and severity of treatment-emergent and serious adverse events		Ongoing 5-year active-treatment study designed to assess the long-term safety and efficacy of mavacamten. The cohort of EXPLORER-LTE confirmed mavacamten’s sustained efficacy with durable LVOT gradient, NT-proBNP levels and NYHA class reduction. Mavacamten treatment was well tolerated over a median 62-week follow-up.

*CPET* cardiopulmonary exercise testing, *HCM* hypertrophic cardiomyopathy, *HCMSQ* Hypertrophic Cardiomyopathy Symptom Questionnaire, *oHCM* obstructive hypertrophic cardiomyopathy, *KCCQ* Kansas City Cardiomyopathy Questionnaire, *KCCQ-CSS* Kansas City Cardiomyopathy Questionnaire – Clinical Summary Score, *KCCQ-OSS* Kansas City Cardiomyopathy Questionnaire – Overall Summary Score, *LAVI* left atrial volume index, *LVEF* left ventricular ejection fraction, *LVOT* left ventricular outflow tract, *NT-proBNP* N-terminal pro B-type natriuretic peptide, *NYHA* New York Heart Association, *pVO₂ *peak oxygen consumption, *SRT* septal reduction therapy, *SpO₂ *peripheral oxygen saturation

The phase 3 EXPLORER-HCM trial (NCT03470545) was a multicenter, double-blind, placebo-controlled study including 251 patients with symptomatic oHCM (NYHA class II–III, LVEF ≥ 55%) and LVOT gradient ≥ 50 mmHg [25]. Patients were randomized 1:1 to mavacamten (2.5–15 mg) or placebo for 30 weeks, on top of beta-blockers or calcium channel blockers (disopyramide excluded). The composite primary endpoint - improvement in pVO₂ and NYHA class - was met in 37% of mavacamten-treated patients versus 17% in the placebo group (*p* = 0.0005) [25]. Key secondary endpoints also favored mavacamten compared to placebo: significant reductions in resting and post-exercise LVOT gradients (−36 mm Hg, 95% confidence interval [CI] −43 to −28; *p* < 0.0001), improved quality of life scores, and lower levels of N-terminal prohormone of brain natriuretic peptide (NT-proBNP) and high-sensitivity troponin I. A complete clinical response (NYHA class I and post-exercise gradient < 30 mmHg) was achieved in 27.4% of treated patients, compared with 0.8% in the placebo group. LVEF decreased modestly (mean − 4%), with reversible reductions < 50% occurring in 5.7% of cases [25]. EXPLORER-CN (NCT05174416), the Chinese arm of the EXPLORER program, enrolled 81 patients with oHCM and LVEF ≥ 55% and confirmed mavacamten efficacy in this population. Notably, over 80% of patients receiving mavacamten achieved a resting or post-exercise LVOT gradient < 50 mmHg, with 56% reaching < 30 mmHg [26].

The VALOR-HCM trial (NCT04349072) investigated whether mavacamten could reduce the need for SRT in symptomatic oHCM patients referred for invasive intervention [27]. A total of 112 patients with NYHA class III–IV symptoms and LVOT gradient ≥ 50 mmHg at rest/provocation were randomized to mavacamten or placebo for 16 weeks. The primary endpoint - proceeding to or remaining eligible for SRT - was met in only 17.9% of mavacamten-treated patients versus 76.8% in the placebo group (*p* < 0.001). Mavacamten also led to greater reductions in post-exercise LVOT gradient (–37 mmHg), improved functional class (≥ 1 NYHA class in 63%), and lower NT-proBNP and troponin I levels, alongside better patient-reported outcomes [27]. The long-term extension of VALOR-HCM, with follow-up to 128 weeks, confirmed sustained benefits: only 15.7% of patients ultimately met SRT criteria, while 80.5% improved by ≥ 1 NYHA class, and most patients (88%) transitioned to commercial therapy [28].

MAVA-LTE (NCT03723655) is an ongoing long-term extension study enrolling patients from the EXPLORER-HCM trial. Interim results confirmed the sustained efficacy of mavacamten over a median follow-up of 62 weeks, with durable reductions in resting and Valsalva LVOT gradients (–35.6 mmHg and − 45.3 mmHg, respectively), significant decreases in NT-proBNP levels, and improvement in NYHA class in over two-thirds of patients [29]. The safety profile remained favorable, with transient LVEF reductions < 50% in a small subset of patients that resolved upon temporary treatment interruption [29].

In the non-obstructive HCM (noHCM) setting, MAVERICK-HCM (NCT03442764), a phase 2 double-blind randomized trial, enrolled 59 patients with LVEF ≥ 55%, NT-proBNP ≥ 300 pg/ml, and NYHA class II–III symptoms [30]. Participants were randomized to mavacamten or placebo for 16 weeks. Mavacamten was associated with significant reductions in NT-proBNP and high-sensitivity troponin levels [30]. Building on this, the phase 3 ODYSSEY-HCM trial (NCT05582395) enrolled 580 symptomatic patients with noHCM to assess whether mavacamten could improve exercise capacity (pVO_2_) and health status (i.e. Kansas City Cardiomyopathy Questionnaire clinical summary score [KCCQ-CSS]) at 48 weeks [31]. However, the trial did not meet either of its dual primary endpoints [86]. While disappointing, these results underscore the biological and clinical differences between obstructive and nonobstructive HCM and suggest that alternative or more tailored therapeutic approaches may be needed for the latter population.

Finally, a phase 3 pediatric trial (NCT06253221) will evaluate mavacamten in symptomatic oHCM children aged 12 to 17 years to define its therapeutic potential across broader age groups and stages of disease.

### Subanalyses and Specialized Outcomes

#### Age, Sex, and Genetic Status

Several exploratory and prespecified analyses have examined whether demographic and genetic factors influence the clinical response to mavacamten. In the EXPLORER-HCM trial, predictably, patients over 60 years or with a diagnosis > 5 years had more comorbidities, lower pVO₂, and higher NT-proBNP levels compared to younger or more recently diagnosed patients. Nonetheless, all groups experienced similar improvements in exercise capacity, NYHA functional class, and patient-reported outcomes, indicating that mavacamten’s efficacy is maintained regardless of age or disease duration [32]. Similarly, the magnitude of benefit from mavacamten was comparable in those with and without hypertension across all endpoints [33].

Sex-based differences were also evaluated. At baseline, women in the EXPLORER-HCM trial presented with a more symptomatic profile than men, including lower pVO₂, higher NT-proBNP, and worse health status scores [34]. Despite this, the treatment effect of mavacamten was similar in men and women for primary and secondary outcomes, while women showed greater improvement in patient-reported health status and NT-proBNP reduction [34]. These findings were confirmed in the long-term VALOR-HCM extension, where women maintained similar sustained clinical benefits over 128 weeks, including reduced eligibility for SRT and stable safety profiles [35].

Preclinical evidence suggests that the response to mavacamten may depend on the specific genetic variant involved. In a study by Ochala et al., thick filament variants were associated with impaired myosin super-relaxation and a favorable in vitro response to mavacamten, whereas thin filament variants preserved the SRX state and showed a blunted pharmacological effect [36]. To date, the only clinical study addressing this issue is a subanalysis of the VALOR-HCM trial, which showed that long-term treatment responses to mavacamten were similar regardless of the presence or absence of sarcomere gene variants [37]. However, this analysis did not differentiate between individual gene variants. In the future, a more granular genetic approach could help identify patients most likely to benefit from CMIs.

#### Electrophysiological Effects

Mavacamten is associated with dynamic and progressive changes in the surface electrocardiogram (ECG) of patients with oHCM. Classical HCM patterns - including left ventricular hypertrophy, prominent Q waves, and T-wave inversions - can attenuate significantly during treatment. Case-based observations have documented stepwise normalization of ECG findings with mavacamten, followed by reappearance of HCM patterns upon treatment withdrawal, and subsequent resolution after reinitiation [38]. These changes closely mirror fluctuations in LVOT gradients, LV function, and symptom burden, suggesting a mechanistic link between structural unloading and electrical remodeling [38]. In support of this, artificial intelligence (AI)-enhanced ECG analysis from the PIONEER-OLE study showed that HCM diagnostic scores - derived from deep learning algorithms trained to recognize HCM-specific features - progressively declined during mavacamten therapy [39]. This may indicate a possible loss of the “electrical fingerprint” of the disease following mavacamten treatment, also highlighting AI-ECG as a promising tool for longitudinal monitoring of therapeutic response.

Data from MAVA-LTE study demonstrated a substantial reduction in mechanical dispersion (median 110 to 59 ms) and QT dispersion (80 to 40 ms), two well-established markers of arrhythmic risk in HCM [40]. These changes were paralleled by clear attenuation of repolarization abnormalities, including resolution of T-wave inversions and ST-segment depressions on surface ECG. Notably, global longitudinal strain (GLS) remained stable throughout follow-up, indicating that improvements in electromechanical synchrony occurred without compromising systolic function, and supporting the hypothesis that mavacamten may contribute to myocardial electrical stabilization [40]. A separate observational study evaluated arrhythmia burden using serial Holter monitoring in patients treated with mavacamten [41]. While short-term treatment was associated with transient increases in premature atrial and ventricular contractions, no sustained or high-risk ventricular arrhythmias were observed, and arrhythmic indices returned to baseline over time [41].

In RCTs, atrial fibrillation (AF) was reported as an adverse event in approximately 2–7% of participants treated with mavacamten, with no significant differences between treatment and placebo arms [42]. However, data from real-world registries suggest a higher baseline prevalence of AF in clinical practice (20–30%) and an incidence of new or progressive AF during follow-up of around 11% [43, 44]. Despite this, AF was generally well controlled through a combination of antiarrhythmic therapy, catheter ablation, and optimized rate control. Only isolated cases required temporary interruption of mavacamten, and permanent discontinuation was rare [45]. In another single, real-world cohort, mavacamten did not increase the risk of new AF and appeared to reduce the need for cardioversion in those with a history of AF [46]. These findings underscore the importance of structured rhythm monitoring and early intervention strategies, particularly in high-risk patients or those with a prior history of AF.

#### Cardiac Imaging and Remodeling

A growing body of imaging evidence confirms that mavacamten induces reverse remodeling in HCM, with favorable effects on diastolic function, atrial and ventricular structure, and myocardial strain.

In the cardiac magnetic resonance (CMR) substudy of EXPLORER-HCM, 30 weeks of mavacamten therapy led to a substantial reduction in LV mass index (–17 vs. − 1.6 g/m² with placebo; *p* < 0.0001), maximum wall thickness, and left atrial volume index (LAVI) (–10.3 mL/m² vs. placebo; *p* = 0.0004). These structural changes occurred without significant changes in late gadolinium enhancement or extracellular volume fraction. Importantly, contractile function remained preserved, with no patient experiencing LVEF < 50% by CMR [47].

Serial strain imaging in VALOR-HCM further addressed safety concerns about potential systolic impairment. At baseline, LV-GLS was already reduced (–14.7%), but improved significantly after 56 weeks of mavacamten therapy [48]. In patients who experienced a transient drop in LVEF < 50%, baseline LV-GLS was markedly worse (–11.4%) but did not decline further during follow-up. Right ventricular strain parameters remained unchanged [48]. In parallel, a separate analysis from VALOR-HCM demonstrated improvements in LA strain indices over 56 weeks of treatment, alongside a significant mean LAVI reduction of − 5.7 mL/m². These changes were attenuated in patients with a history of AF, suggesting that atrial myopathy may limit remodeling capacity in that subgroup [49].

Echocardiographic data from EXPLORER-HCM and EXPLORER-CN reinforce these findings. In EXPLORER-HCM, mavacamten reduced lateral E/e′ by − 3.8 points (vs. 0.04 with placebo; *p* < 0.0001) and LAVI by − 7.5 mL/m² (vs. − 0.09; *p* < 0.0001), with strong correlations to NT-proBNP reduction [50]. In EXPLORER-CN, similar improvements were observed in lateral and septal E/e′ and LAVI, confirming consistency across populations [51]. Subgroup analyses suggest that these remodeling effects extend even to high-risk individuals, such as those with elevated baseline LV filling pressures (E/e′ ≥15) [52]. Finally, in the VALOR-HCM echocardiographic substudy, almost one-third of patients on mavacamten improved their diastolic function, with a mean E/e′ and LAVI reduction of − 3.4 and − 5.2 mL/m², respectively [53].

The ongoing MEMENTO-HCM study (NCT06112743) is specifically designed to assess the effects of mavacamten on myocardial structure using serial CMR, and will provide further insights into its potential as a disease-modifying therapy in oHCM.

#### Health and Functional Status

Mavacamten has demonstrated consistent and clinically meaningful benefits across multiple domains of patient-reported outcomes and functional performance. In the EXPLORER-HCM trial, after 30 weeks of treatment, patients receiving mavacamten showed a significantly greater improvement in KCCQ Overall Summary (OS) score compared to placebo (mean change + 14.9 vs. +5.4; *p* < 0.0001) [54]. Notably, 36% of mavacamten-treated patients achieved a ≥ 20-point increase, corresponding to a number needed to treat of just 5 for a large clinical improvement. Benefits were consistent across all KCCQ subscales - symptom burden, physical and social limitations, and quality of life - and returned to baseline upon drug washout [54].

In parallel, exploratory cardiopulmonary exercise testing data from EXPLORER-HCM demonstrated objective improvements in both maximal and submaximal exercise performance. Compared with placebo, mavacamten significantly increased peak metabolic equivalents (mean difference + 0.4; *p* < 0.001), peak circulatory power (+ 373 mL/kg/min·mmHg; *p* = 0.001), and peak end-tidal CO₂ (+ 2.0 mmHg; *p* < 0.001) [55]. Improvements were also seen in peak exercise time and ventilatory efficiency, with a lower VE/VCO₂ slope and higher ventilatory power. These results suggest a broader effect on cardiovascular and ventilatory performance beyond pVO₂ max alone [55].

## Real-world Evidence

Following the promising results coming from clinical trials, mavacamten has been approved for the treatment of symptomatic oHCM by the United States (US) Food and Drug Administration (FDA) in April 2022 and by the European Medicines Agency (EMA) in 2023. As a consequence, it has been incorporated into both the 2023 European Society of Cardiology (ESC) and 2024 American College of Cardiology/American Heart Association (ACC/AHA) guidelines as a second-line pharmacologic option for patients with oHCM who remain symptomatic despite medical treatment [56, 57]. Specifically, the ESC guidelines recommend either disopyramide (I B) or mavacamten (IIa A), or even mavacamten as monotherapy for patients who are intolerant or have contraindications to beta-blockers, verapamil/diltiazem, or disopyramide (IIa B) [56]. Conversely, the American guidelines propose the following second-line therapies with the same class and level of evidence: a CMI (therefore including also aficamten), disopyramide, or SRT (1 B) [57, 58].

These developments have prompted a growing body of real-world evidence, which will be discussed in terms of patient candidacy, clinical efficacy, dosing strategies, safety monitoring, and cost.

### Real-world Candidacy

In RCTs, eligibility for mavacamten required NYHA class II–III symptoms, LVEF >55%, and a peak LVOT gradient >30 mmHg despite optimized background therapy. However, multiple real-world registries suggest that these inclusion criteria apply to only a subset of patients routinely seen in oHCM clinics. For instance, Amr et al. found that just 10% of their HCM cohort met EXPLORER-HCM criteria, with the LVOT gradient threshold being the most common limiting factor [59]. In the Bertero et al. multicenter study across Italy, 49% of patients with obstructive physiology and persistent symptoms were considered eligible for mavacamten under ESC guidelines [60]. Similar proportions were reported by de Gregorio (47%) and Parodi (31%) in national registries from Italy and France, respectively [61, 62]. Notably, real-world patients were generally older, had more AF, and showed more advanced structural disease.

### Efficacy in Practice

The clinical effectiveness of mavacamten has been corroborated by a growing number of real-world studies across a range of geographic regions, patient populations, and healthcare settings (Table 2). In real-world practice, treatment with mavacamten led to significant reductions in both resting and Valsalva LVOT gradients, typically ranging from 28 to 67 mmHg at rest and 42 to 80 mmHg during Valsalva [43, 44, 63–70]. In the Asiatic cohorts from South Korea and Japan, reductions in Valsalva gradients exceeded 60 mmHg, even in patients with severe baseline obstruction [43, 70].

**Table 2:** Real-world studies of Mavacamten in patients with symptomatic obstructive hypertrophic cardiomyopathy

Center and *N*. of Patients	Baseline Characteristics	Follow-up	Main Outcomes Efficacy	Main Outcomes Safety	Dose Modifications	Ref.
Single center (USA) *n* = 23	Mean age: 61 years; Female: 61%; All on BB or CCB monotherapy	5.3 months (IQR: 81–207 days)	↓ Rest LVOT gradient (↓ 33 mmHg) ↓ Valsalva gradient (↓ 67 mmHg) ↓ E/e’ (19.2→15.5) NYHA class improved in 56% ECG normalization in 21.7%	Well tolerated; No LVEF < 50%; Minor AEs in 4 pts (palpitations *n* = 2, dyspnea *n* = 1, dizziness *n* = 1)	↑ Dose in 6 pts (23%); ↓ Dose in 4 pts (17%); 35% reduced background therapy	*Abdelfattah*,* 2023* [63]
Single center (Germany) *n* = 15	Mean age: 54 years; Female: 40% 87% BB, 6% CCB	3 months	↓ Rest LVOT gradient (↓ 28 mmHg) ↓ Valsalva gradient (↓ 63 mmHg) NYHA class improved in 67% ↓ NT-proBNP levels (↓ 358 pg/ml)	Well tolerated. Stopped in 1 pt (persistent LVEF reduction)	Temporary treatment interruptions occurred in 8 pts (gastrointestinal/ ophthalmological complaints or transient LVEF < 50%) with subsequent full recovery	*Becker*,* 2024* [64]
Single center (USA) *n* = 150	Mean age: 65 years; Female: 53% 83% BB	3 months	↓ Valsalva gradient (↓ 42 mmHg) NYHA class improved in 46% Stable LVEF	Well tolerated	Temporary treatment interruptions occurred in 3 pts	*Desai*,* 2024* [44]
Single center (USA) *n* = 50	Mean age: 63 years; Female: 64% 68% BB, 16% CCB	3 months	↓ Rest LVOT gradient (↓ 33 mmHg) ↓ Valsalva gradient (↓ 51 mmHg); NYHA class significantly improved No significant change in E/e’	Stopped in 2 pts for fatigue/malaise	Mavacamten was temporarily held in 5 pts due to Valsalva LVOT gradient < 20 mmHg, and in 2 pts for LVEF < 50% Dosage: 2.5 mg (20%), 5 mg (38%), 10 mg (29%), and 15 mg (13%)	*Kim*,* 2024* [65]
Multicenter (Japan) HORIZON-HCM *n* = 38	Mean age: 65 years; Female: 66% 89% BB, 8% CCB	30 weeks	↓ Rest LVOT gradient (↓ 61 mmHg) ↓ Valsalva gradient (↓ 71 mmHg) NYHA class improved in 73% ↓ NT-proBNP levels (↓ 738 pg/ml) ↓ E/e’ (6.9) Mild LVEF reduction (−3%)	Mild (37%) or moderate (26%) AEs AF (8%) Stopped in 1 pt due to QTc prolongation	Temporary treatment interruptions occurred in 8 pts	*Kitaoka*,* 2024* [43]
Single center (USA) *n* = 66	Mean age: 59 years; Female: 53% 89% BB, 28% CCB	9 months	↓ Rest LVOT gradient (↓ 37 mmHg) ↓ Valsalva gradient (↓ 80 mmHg) NYHA class improved in 72% Mild but significant LVEF reduction (−7%)	No medication-related AEs	Temporary treatment interruptions occurred in 3 pts Dose: 2.5 mg (21%), 5 mg (42%), 10 mg (35%), and 15 mg (2%)	*Ramonfaur*,* 2024* [66]
Single-center (USA) *n* = 96	Mean age: 63 years; Female: 54% 55% BB	9 months	↓ Rest LVOT gradient ↓ Valsalva gradient NYHA class improved in 60% LVEF reduction (−7%)	Well tolerated	Temporary treatment interruptions occurred in 2 pts Dose: 2.5 mg (9%), 5 mg (46%), 10 mg (43%), and 15 mg (2%)	*Reza*,* 2024* [67]
Single center (USA) *n* = 61	Mean age: 57 years Female: 52% 95% BB, 34% CCB, and 11% disopyramide	6 months	↓ Rest LVOT gradient (↓ 67 mmHg) Stable LVEF and GLS over time ↑ Grade 1 diastolic dysfunction (27% → 62%)	Well tolerated	Temporary treatment interruptions occurred in 1 pts	*Abood*,* 2025* [68]
Three centers (UK) *n* = 93	Mean age: 60 years; Female: 28% male 78% BB, 18% CCB, 37% disopyramide, 5% prior SRT	3 months	↓ Rest LVOT gradient (↓ 30 mmHg); ↓ Valsalva gradient (↓ 45 mmHg); NYHA class improved in 74% ↓ NT-proBNP levels (↓ 518 pg/ml)	Well tolerated	Temporary treatment interruptions occurred in 13 pts Dose was 2.5 mg in 17%, 5 mg in 32%, 10 mg in 43% and 15 mg in 7% of patients. The average time to treatment optimisation was 15.8 weeks.	*Burford*,* 2025* [69]
Multicenter (South Korea) *n* = 46	Mean age: 62 years; Female: 44% male 76% BB, 15% CCB, 37% disopyramide, 5% prior SRT	147 days (IQR 56–205)	↓ Rest LVOT gradient (↓ 41 mmHg); ↓ Valsalva gradient (↓ 67 mmHg); NYHA class improved in 58% ↓ NT-proBNP levels (↓ 693 pg/ml) Mild LVEF reduction (−3%) ↓ MWT (−1.8 mm) and ↓ LAVI (−6.5 ml/m^2^)	Well tolerated Stopped in 1 case (AF and LVEF reduction)	Temporary treatment interruptions occurred in 14 pts Dose: ≤5 (75%) and 10 mg (25%)	*Lim*,* 2025* [70]

*AF* atrial fibrillation, *AEs* adverse events, *BB* beta-blocker, *CCB* calcium channel blocker, *ECG* electrocardiogram, *E/e′* mitral inflow velocity to annular early diastolic velocity ratio, *GLS* global longitudinal strain, *HCM* hypertrophic cardiomyopathy, *oHCM* obstructive hypertrophic cardiomyopathy,* IQR* interquartile range, *KCCQ* Kansas City Cardiomyopathy Questionnaire, *LAVI* left atrial volume index, *LV *left ventricle, *LVEF* left ventricular ejection fraction, *LVOT *left ventricular outflow tract, *MWT* maximal wall thickness, *NT-proBNP* N-terminal pro-brain natriuretic peptide, *NYHA* New York Heart Association *pts* patients, *QTc* corrected QT interval, *SRT* septal reduction therapy

Functional improvements were also consistently reported. NYHA class improved of at least 1 class in 46–74% of patients, with the highest rates of symptomatic benefit observed in multicenter cohorts and those treated for longer durations [67, 69]. Importantly, these improvements were seen despite variability in background therapy and patient comorbidities, supporting the additive value of mavacamten when used in routine practice.

Biomarker responses paralleled the clinical benefits. Reductions in NT-proBNP levels were frequently reported, in some cases exceeding 700 pg/mL [43]. In some studies, echocardiographic data also showed improvements in septal thickness and LAVI, suggesting structural reverse remodeling in addition to symptomatic relief [70].

Importantly, real-world data also support the use of mavacamten in patients who had previously undergone but failed SRT. In the study by Abood et al., all eight patients with prior unsuccessful SRT experienced marked improvement with mavacamten. The mean provoked LVOT gradient decreased from 69 to 9 mmHg at 6 months, with all patients improving in NYHA class and six becoming asymptomatic (class I) [68].

### Dose Titration and Most Commonly Used Regimens

Mavacamten is typically initiated at a dose of 5 mg once daily. Dose adjustments are made every 4 to 8 weeks based on LVOT gradient and LVEF, using available doses of 2.5, 5, 10, or 15 mg/day. In real-world practice, the most common maintenance dose is 10 mg (43% of patients), followed by 5 mg (32%), with lower (2.5 mg) or higher (15 mg) doses used less frequently [69]. The average time to dose optimization ranges from 12 to 16 weeks [69, 70]. Dose escalation is often required for suboptimal gradient reduction [63], whereas dose reductions may occur in response to asymptomatic LVEF decline or minor side effects [64, 70]. Overall, most patients achieve a stable long-term regimen after initial titration, with good tolerability and without need for permanent discontinuation in the majority of cases [63, 64, 69].

### Side Effects and Monitoring

Due to its nature of negative inotrope, mavacamten carries a risk of systolic dysfunction and has been made available in the US exclusively through the CAMZYOS REMS Program (https://www.camzyosrems.com). This program mandates certification of prescribers and pharmacies, patient enrollment, and regular echocardiographic monitoring to ensure safe dose titration and identify early signs of reduced LVEF. In the EXPLORER-HCM trial, 6% of patients in the mavacamten arm experienced reversible reductions in LVEF < 50%. Notably, syncope (0.8%) was the only adverse event that led to drug discontinuation. Most adverse events were mild, with dizziness and syncope being more frequent than in the placebo group.

In real-world use, REMS surveillance data on 1,866 patients showed consistent findings: 2.8% had LVEF reduction < 50%, and 1.1% experienced heart failure (HF) requiring hospitalization [71]. A similar pharmacovigilance study using the FAERS database identified reduced LVEF, HF, AF, and dizziness as the most significant adverse drug reactions (ADRs) [72].

In real-world cohorts, LVEF remained stable in most patients, with occasional mild reductions (< 10%) that were generally asymptomatic and reversible after dose adjustment or temporary interruption [68]. Permanent discontinuation was rare, and no cases of sustained systolic dysfunction or LVEF < 30% were reported [69]. Notably, GLS remained preserved or improved, supporting the safety of mavacamten on systolic function [68]. Transient adverse effects such as dizziness, hypotension, and mild LVEF decline were the most common, while new but uncommon events - such as peripheral edema, urinary tract infection, and gout - have emerged and warrant further investigation [69].

Overall, the safety profile of mavacamten in clinical practice appears consistent with RCTs, reinforcing the importance of structured monitoring, particularly during the early titration phase.

### Pharmacogenetic Influence and Drug-drug Interactions

Mavacamten is primarily metabolized by CYP2C19 (74%), with additional contribution from CYP3A4 (18%) and CYP2C9 (8%) [73]. As such, concomitant use with strong CYP2C19 inhibitors (e.g., fluvoxamine) or moderate to strong inducers of CYP2C19 or CYP3A4 is contraindicated due to the elevated risk of excessive exposure or therapeutic failure. Co-administration with moderate inhibitors (e.g., verapamil or omeprazole) requires careful monitoring and potential dose adjustments [74]. On the contrary, mavacamten can be taken with or without food, as food does not significantly affect its absorption [75].

Pharmacogenetics plays a critical role in mavacamten safety and efficacy. CYP2C19 poor metabolizers - who carry two no-function alleles - exhibit significantly increased drug exposure, with a 241% increase in area under the curve and 47% increase in Cmax compared to normal metabolizers, alongside a prolonged half-life (23 vs. 6–9 days) [73]. These individuals are at increased risk of systolic dysfunction, even at standard doses. The prevalence of poor metabolizers varies: ~2% in Europeans, ~ 13% in East Asians, and up to 57% in Oceanic populations. The EMA recommend *CYP2C19* genotyping prior to initiating therapy, with reduced initial dosing (2.5 mg daily, max 5 mg) in poor metabolizers.

### Cost-effectiveness

The cost-effectiveness of mavacamten has been evaluated through modeling studies and health technology assessments. A French health economic analysis estimated that adding mavacamten to standard therapy for symptomatic oHCM resulted in an incremental cost-effectiveness ratio (ICER) of €52,903 per Quality-Adjusted Life Year (QALY) gained, compared to standard care alone [76]. Mavacamten may therefore be a cost-effective option, particularly when used in patients who are not eligible for SRT.

According to the UK’s National Institute for Health and Care Excellence (NICE), mavacamten is recommended as an add-on to optimized standard therapy for adults with oHCM who remain severely symptomatic, provided that REMS monitoring is feasible. NICE’s cost-effectiveness analysis reported an ICER of £21,900 per QALY gained, which falls within the accepted cost-effectiveness threshold in the UK.

In the US, a separate evaluation conducted by the Institute for Clinical and Economic Review modeled a lifetime horizon and projected a cost per QALY gained ranging from $150,000 to $200,000 depending on scenario assumptions. The Institute report raised concerns about affordability at list price, while recognizing the potential to reduce downstream costs associated with SRT and HF-related complications. On this point, Desai et al. performed a US-based modeling study using data from EXPLORER and VALOR-HCM. They reported that mavacamten reduced the need for invasive therapies and improved QALY, with an ICER below commonly accepted US willingness-to-pay thresholds in several modeled subgroups [77].

## Evolving Role of Myosin Inhibitors Versus Standard Therapy in Obstructive HCM

The introduction of CMIs has redefined the therapeutic landscape of oHCM. Until recently, symptom relief relied almost exclusively on beta-blockers or non-dihydropyridine calcium channel blockers, with disopyramide reserved as a second-line option or as a step prior to SRT.

Subgroup analyses from EXPLORER-HCM have shown that mavacamten exerts consistent benefits on LVOT gradients, symptoms, and biomarkers irrespective of background beta-blocker therapy [78]. However, patients receiving concomitant beta-blockers exhibited high rates of chronotropic incompetence - over 90% in EXPLORER-HCM - which translated into attenuated improvements in heart rate–dependent parameters such as peak oxygen uptake, exercise time, and maximal heart rate. By contrast, heart rate–independent measures, including VE/VCO₂ slope, NT-proBNP, and LVOT gradients, improved similarly in patients with or without beta-blockade [78]. These findings indicate that while beta-blockers do not blunt the hemodynamic effects of mavacamten, they may limit its impact on exercise capacity and their withdrawal might unmask even greater functional gains [79].

Disopyramide, historically considered the most potent negative inotrope for oHCM, has demonstrated efficacy in reducing LVOT gradients and deferring SRT in long-term observational studies [80]. Nonetheless, its widespread use has been limited by anticholinergic side effects and occasional pro-arrhythmic risk. Importantly, disopyramide was excluded from pivotal mavacamten trials, though it was permitted in VALOR-HCM, where only a minority of patients received it and no formal interaction analysis was undertaken. Regulatory guidance continues to discourage concomitant administration of disopyramide and CMIs because of the potential for additive negative inotropy. More recently, case series and clinical practice reports have described safe transition protocols from disopyramide to mavacamten [81]. A stepwise tapering of disopyramide while initiating mavacamten appeared safer and more effective than abrupt washout, preventing symptom worsening and avoiding adverse effects during the transition [81].

Beyond mavacamten, aficamten represents the second-in-class CMI, characterized by a shorter half-life (approximately 3–6 days) and more rapid onset of action [3]. The SEQUOIA-HCM trial confirmed its efficacy and safety in 282 patients with symptomatic oHCM, demonstrating significant reductions in LVOT gradients, improvements in NYHA class, KCCQ score, and NT-proBNP levels, and low rates of systolic dysfunction over 24 weeks [82]. Building on these findings, the recently published MAPLE-HCM trial provided the first direct head-to-head comparison between a CMI and a conventional agent. In 175 patients randomized to aficamten or metoprolol monotherapy, aficamten was superior across multiple endpoints, including peak oxygen uptake, NYHA class, health status, LVOT gradient, NT-proBNP, and left atrial volume index, with a safety profile comparable to metoprolol [83]. MAPLE-HCM therefore suggests that CMIs may not only complement, but in selected cases even replace, traditional agents such as beta-blockers as initial therapy for oHCM [83].

Taken together, these data support a shift away from a rigid treatment sequence in which CMIs are introduced only after conventional therapies fail. Conventional agents continue to play a role, particularly in mildly symptomatic patients or where CMIs are unavailable, but the balance is increasingly moving toward disease-specific therapies that directly target sarcomeric hypercontractility. Future studies will be crucial to define the role of monotherapy with CMIs, the safety and efficacy of withdrawing background negative inotropes, and the optimal strategy for transitioning patients from disopyramide to this new class of targeted drugs.

## Expanding Indications and Future Directions

CMIs have opened unprecedented therapeutic avenues in HCM. While both mavacamten and aficamten have demonstrated robust efficacy in oHCM, future research will need to clarify their role across broader patient populations and disease stages. Beyond their use in symptomatic obstructive disease, CMIs are being investigated in conditions characterized by sarcomeric hypercontractility, including non-obstructive HCM, HF with preserved EF (HFpEF), and early-stage or genotype-positive/phenotype-negative cohorts, where early intervention could theoretically delay or prevent disease progression [84, 85].

The recently published results of ODYSSEY-HCM, which failed to confirm the benefits of CMIs in non-obstructive HCM despite encouraging phase 2 data, underscore the need for a more nuanced understanding of disease biology and patient stratification [86]. Similarly, pediatric HCM remains an important unmet area where dedicated clinical trials are underway. In parallel, novel molecules are being developed. EDG-7500 is a novel cardiac sarcomere modulator that destabilizes actomyosin cross-bridges during low calcium conditions, enhancing relaxation without reducing myosin motor function; early-phase trials showed good tolerability and support once-daily dosing [87].

Another key challenge is the marked anatomic and phenotypic heterogeneity of HCM. Real-world experience has shown that a subset of patients - particularly those with apical or mid-cavity obstruction or concomitant mitral valve anomalies - may derive limited benefit from CMIs [88]. Advanced imaging modalities, particularly CMR, together with artificial intelligence–based tools, hold promise in refining patient selection and predicting therapeutic response.

Finally, long-term data from MAVA-LTE suggest that sustained treatment with mavacamten provides durable improvements in gradients, symptoms, and biomarkers, with an acceptable safety profile [29]. Whether CMIs can ultimately modify the natural history of HCM by preventing fibrosis, reducing arrhythmic risk, and lowering the incidence of SCD remains a central and unanswered question. Addressing this will require longer follow-up, larger real-world registries, and continued integration of genetic and imaging markers into clinical decision-making.

## Conclusions

Mavacamten represents a major advance in the treatment of oHCM, targeting the disease at its molecular core. By modulating sarcomeric hypercontractility, it effectively reduces LVOT gradients, improves symptoms, and induces structural remodeling. Clinical trial data have been confirmed by real-world evidence, supporting its safety and efficacy across diverse populations. Ongoing research will clarify its role in pediatric, early-stage, and anatomically complex HCM, as well as its potential in other hypercontractile phenotypes. As experience grows, optimized patient selection and precision dosing will be key to maximizing benefits while minimizing risks in clinical practice.

AF, atrial fibrillation; AEs, adverse events; BB, beta-blocker; CCB, calcium channel blocker; ECG, electrocardiogram; E/e′, mitral inflow velocity to annular early diastolic velocity ratio; GLS, global longitudinal strain; HCM, hypertrophic cardiomyopathy; oHCM, obstructive hypertrophic cardiomyopathy; IQR, interquartile range; KCCQ, Kansas City Cardiomyopathy Questionnaire; LAVI, left atrial volume index; LV, left ventricle; LVEF, left ventricular ejection fraction; LVOT, left ventricular outflow tract; MWT, maximal wall thickness; NT-proBNP, N-terminal pro-brain natriuretic peptide; NYHA, New York Heart Association; pts, patients; QTc, corrected QT interval; SRT, septal reduction therapy.

## Key References

- Rader F, Oręziak A, Choudhury L, et al (2024) Mavacamten Treatment for Symptomatic Obstructive Hypertrophic Cardiomyopathy: Interim Results From the MAVA-LTE Study, EXPLORER-LTE Cohort. JACC Heart Fail 12:164–17.
  - This interim analysis from the EXPLORER cohort of the MAVA-LTE study represents the largest and longest report of mavacamten use in oHCM.
- Desai MY, Hajj-Ali A, Rutkowski K, Ospina S, Gaballa A, Emery M, Asher C, Xu B, Thamilarasan M, Popovic ZB (2024) Real-world experience with mavacamten in obstructive hypertrophic cardiomyopathy: Observations from a tertiary care center. Prog Cardiovasc Dis 86:62–68.
  - This is the largest real-world experience in a HCM tertiary care center published so far.
- Martinez MW, Seto D, Cheung M, Coiro M, Patel N, Bastien A, Lockman J, Afsari S, Desai MY (2025) Mavacamten: Initial Insights From the Risk Evaluation and Mitigation Strategy Program. JACC Adv 4:101430.
  - The first comprehensive safety data from the REMS program, demonstrating low rates of serious adverse events and supporting safe outpatient use of mavacamten.

## Data Availability

No datasets were generated or analysed during the current study.

## References

[1] Maron MS, Olivotto I, Zenovich AG, Link MS, Pandian NG, Kuvin JT, et al. Hypertrophic cardiomyopathy is predominantly a disease of left ventricular outflow tract obstruction. Circulation. 2006;114:2232–9. 17088454 10.1161/CIRCULATIONAHA.106.644682

[2] Braunwald E, Saberi S, Abraham TP, Elliott PM, Olivotto I. Mavacamten: a first-in-class myosin inhibitor for obstructive hypertrophic cardiomyopathy. Eur Heart J. 2023;44:4622–33. 37804245 10.1093/eurheartj/ehad637 PMC10659958

[3] Davis BJ, Volk H, Nguyen O, et al. Safety and efficacy of Mavacamten and Aficamten in patients with hypertrophic cardiomyopathy. J Am Heart Association. 2025;14:e038758. 10.1161/JAHA.124.038758 PMC12132771 40055855

[4] Ostrominski JW, Guo R, Elliott PM, Ho CY. Cardiac myosin inhibitors for managing obstructive hypertrophic cardiomyopathy. JACC: Heart Failure. 2023;11:735–48. 37407153 10.1016/j.jchf.2023.04.018

[5] Toepfer CN, Garfinkel AC, Venturini G, et al. Myosin sequestration regulates sarcomere function, cardiomyocyte energetics, and metabolism, informing the pathogenesis of hypertrophic cardiomyopathy. Circulation. 2020;141:828–42. 31983222 10.1161/CIRCULATIONAHA.119.042339 PMC7077965

[6] Palandri C, Santini L, Argirò A, Margara F, Doste R, Bueno-Orovio A, et al. Pharmacological management of hypertrophic cardiomyopathy: from bench to bedside. Drugs. 2022;82:889–912. 35696053 10.1007/s40265-022-01728-w PMC9209358

[7] Anderson RL, Trivedi DV, Sarkar SS, et al. Deciphering the super relaxed state of human β-cardiac myosin and the mode of action of Mavacamten from myosin molecules to muscle fibers. Proc Natl Acad Sci U S A. 2018;115:E8143-52. 30104387 10.1073/pnas.1809540115 PMC6126717

[8] Cail RC, Baez-Cruz FA, Winkelmann DA, Goldman YE, Michael Ostap E. (2024) Dynamics of β-cardiac myosin between the super-relaxed and disordered-relaxed States. bioRxiv 2024.12.14.628474. 10.1016/j.jbc.2025.108412 PMC12023885 40118457

[9] Hooijman P, Stewart MA, Cooke R. A new state of cardiac myosin with very slow ATP turnover: a potential cardioprotective mechanism in the heart. Biophys J. 2011;100:1969–76. 21504733 10.1016/j.bpj.2011.02.061 PMC3077696

[10] Daniels MJ, Fusi L, Semsarian C, Naidu SS. Getting inside the engine - myosin modulation in hypertrophic cardiomyopathy and systolic heart failure. Circulation. 2021;144:759–62. 34491773 10.1161/CIRCULATIONAHA.121.056324 PMC7611636

[11] McNamara JW, Li A, Lal S, Bos JM, Harris SP, van der Velden J, et al. MYBPC3 mutations are associated with a reduced super-relaxed state in patients with hypertrophic cardiomyopathy. PLoS ONE. 2017;12:e0180064. 28658286 10.1371/journal.pone.0180064 PMC5489194

[12] Toepfer CN, Wakimoto H, Garfinkel AC, et al. Hypertrophic cardiomyopathy mutations in MYBPC3 dysregulate myosin. Sci Transl Med. 2019;11:eaat1199. 30674652 10.1126/scitranslmed.aat1199 PMC7184965

[13] Alamo L, Ware JS, Pinto A, Gillilan RE, Seidman JG, Seidman CE, et al. Effects of myosin variants on interacting-heads motif explain distinct hypertrophic and dilated cardiomyopathy phenotypes. Elife. 2017;6:e24634. 28606303 10.7554/eLife.24634 PMC5469618

[14] Adhikari AS, Kooiker KB, Sarkar SS, Liu C, Bernstein D, Spudich JA, et al. Early-onset hypertrophic cardiomyopathy mutations significantly increase the velocity, force, and actin-activated ATPase activity of human β-cardiac myosin. Cell Rep. 2016;17:2857–64. 27974200 10.1016/j.celrep.2016.11.040 PMC11088367

[15] Green EM, Wakimoto H, Anderson RL, et al. A small-molecule inhibitor of sarcomere contractility suppresses hypertrophic cardiomyopathy in mice. Science. 2016;351:617–21. 26912705 10.1126/science.aad3456 PMC4784435

[16] Kawas RF, Anderson RL, Ingle SRB, Song Y, Sran AS, Rodriguez HM. A small-molecule modulator of cardiac myosin acts on multiple stages of the myosin chemomechanical cycle. J Biol Chem. 2017;292:16571–7. 28808052 10.1074/jbc.M117.776815 PMC5633120

[17] Robinson P, Liu X, Sparrow A, Patel S, Zhang Y-H, Casadei B, et al. Hypertrophic cardiomyopathy mutations increase myofilament Ca2 + buffering, alter intracellular Ca2 + handling, and stimulate Ca2+-dependent signaling. J Biol Chem. 2018;293:10487–99. 29760186 10.1074/jbc.RA118.002081 PMC6036197

[18] Awinda PO, Bishaw Y, Watanabe M, Guglin MA, Campbell KS, Tanner BCW. Effects of Mavacamten on Ca2 + sensitivity of contraction as sarcomere length varied in human myocardium. Br J Pharmacol. 2020;177:5609–21. 32960449 10.1111/bph.15271 PMC7707091

[19] Sewanan LR, Shen S, Campbell SG. Mavacamten preserves length-dependent contractility and improves diastolic function in human engineered heart tissue. Am J Physiol Heart Circ Physiol. 2021;320:H1112-23. 33449850 10.1152/ajpheart.00325.2020 PMC7988756

[20] Awinda PO, Watanabe M, Bishaw Y, Huckabee AM, Agonias KB, Kazmierczak K, et al. Mavacamten decreases maximal force and Ca2 + sensitivity in the N47K-myosin regulatory light chain mouse model of hypertrophic cardiomyopathy. Am J Physiol Heart Circ Physiol. 2021;320:H881-90. 33337957 10.1152/ajpheart.00345.2020 PMC8082789

[21] Sparrow AJ, Watkins H, Daniels MJ, Redwood C, Robinson P. Mavacamten rescues increased myofilament calcium sensitivity and dysregulation of Ca2 + flux caused by thin filament hypertrophic cardiomyopathy mutations. Am J Physiol Heart Circ Physiol. 2020;318:H715-22. 32083971 10.1152/ajpheart.00023.2020 PMC7099453

[22] Mamidi R, Li J, Doh CY, Verma S, Stelzer JE. Impact of the myosin modulator Mavacamten on force generation and cross-bridge behavior in a murine model of hypercontractility. J Am Heart Assoc. 2018;7:e009627. 30371160 10.1161/JAHA.118.009627 PMC6201428

[23] Heitner SB, Jacoby D, Lester SJ, Owens A, Wang A, Zhang D, et al. Mavacamten treatment for obstructive hypertrophic cardiomyopathy: a clinical trial. Ann Intern Med. 2019;170:741–8. 31035291 10.7326/M18-3016

[24] Masri A, Lester SJ, Stendahl JC, Hegde SM, Sehnert AJ, Balaratnam G, et al. Long-term safety and efficacy of Mavacamten in symptomatic obstructive hypertrophic cardiomyopathy: interim results of the PIONEER-OLE study. J Am Heart Assoc. 2024;13:e030607. 38591260 10.1161/JAHA.123.030607 PMC11262496

[25] Olivotto I, Oreziak A, Barriales-Villa R, et al. Mavacamten for treatment of symptomatic obstructive hypertrophic cardiomyopathy (EXPLORER-HCM): a randomised, double-blind, placebo-controlled, phase 3 trial. Lancet. 2020;396:759–69. 32871100 10.1016/S0140-6736(20)31792-X

[26] Tian Z, Li L, Li X, et al. Effect of mavacamten on Chinese patients with symptomatic obstructive hypertrophic cardiomyopathy: the EXPLORER-CN randomized clinical trial. JAMA Cardiol. 2023;8:957–65. 37639259 10.1001/jamacardio.2023.3030 PMC10463173

[27] Desai MY, Owens A, Geske JB, et al. Myosin inhibition in patients with obstructive hypertrophic cardiomyopathy referred for septal reduction therapy. J Am Coll Cardiol. 2022;80:95–108. 35798455 10.1016/j.jacc.2022.04.048

[28] Desai MY, Wolski K, Owens A, et al. Mavacamten in patients with hypertrophic cardiomyopathy referred for septal reduction: week 128 results from VALOR-HCM. Circulation. 2025;151:1378–90. 39556124 10.1161/CIRCULATIONAHA.124.072445 PMC12063683

[29] Rader F, Oręziak A, Choudhury L, et al. Mavacamten treatment for symptomatic obstructive hypertrophic cardiomyopathy: interim results from the MAVA-LTE Study, EXPLORER-LTE cohort. JACC Heart Fail. 2024;12:164–77. 38176782 10.1016/j.jchf.2023.09.028

[30] Ho CY, Mealiffe ME, Bach RG, et al. Evaluation of Mavacamten in symptomatic patients with nonobstructive hypertrophic cardiomyopathy. J Am Coll Cardiol. 2020;75:2649–60. 32466879 10.1016/j.jacc.2020.03.064

[31] Desai MY, Nissen SE, Abraham T, et al. Mavacamten in symptomatic nonobstructive hypertrophic cardiomyopathy: Design, Rationale, and baseline characteristics of ODYSSEY-HCM. JACC Heart Fail. 2025;13:358–70. 39909647 10.1016/j.jchf.2024.11.013

[32] Wang A, Lakdawala NK, Abraham TP, Nilles EK, Wojdyla DM, Owens AT, et al. Association between age or duration of diagnosis in obstructive hypertrophic cardiomyopathy and response to Mavacamten treatment: exploratory analysis of the EXPLORER-HCM trial. J Card Fail. 2025;31:901–11. 39653325 10.1016/j.cardfail.2024.10.449

[33] Wang A, Spertus JA, Wojdyla DM, Abraham TP, Nilles EK, Owens AT, et al. Mavacamten for obstructive hypertrophic cardiomyopathy with or without hypertension: post-hoc analysis of the EXPLORER-HCM trial. JACC Heart Fail. 2024;12:567–79. 37855754 10.1016/j.jchf.2023.07.030

[34] Cresci S, Bach RG, Saberi S, et al. Effect of Mavacamten in women compared with men with obstructive hypertrophic cardiomyopathy: insights from EXPLORER-HCM. Circulation. 2024;149:498–509. 37961906 10.1161/CIRCULATIONAHA.123.065600 PMC11006596

[35] Desai MY, Saberi S, Geske JB, et al. Long-Term response of obstructive hypertrophic cardiomyopathy patients to Mavacamten based on sex: insights from the VALOR-HCM trial. JACC Heart Fail. 2025;13:1037–40. 40100181 10.1016/j.jchf.2025.02.005

[36] Ochala J, Feng M, Wang Q et al. (2025) Heterogeneous dysregulation of myosin Super-Relaxation and energetics in hypertrophic cardiomyopathy. Circ Heart Fail e012614. 10.1161/CIRCHEARTFAILURE.124.012614 PMC12353603 40391807

[37] Desai MY, Owens A, Saberi S et al. (2025) Long-Term effects of Mavacamten on patients based on hypertrophic cardiomyopathy pathogenic genetic variant status: insights from VALOR-HCM trial. Circ Genom Precis Med e005100. 10.1161/CIRCGEN.125.005100 40163785

[38] Austin M, Marzolf A, Hwang I, Puhl J, de Feria A, Reza N, et al. PO-05-054 dynamic impact of Mavacamten on ECG patterns of left ventricular hypertrophy with left ventricular strain. Heart Rhythm. 2024;21:S596.

[39] Siontis KC, Abreau S, Attia ZI, et al. Patient-Level artificial Intelligence–Enhanced electrocardiography in hypertrophic cardiomyopathy. JACC: Advances. 2023;2:100582. 38076758 10.1016/j.jacadv.2023.100582 PMC10702858

[40] Del Franco A, Palinkas ED, Bellagamba CCA, Biagioni G, Zampieri M, Marchi A, et al. Long-term effects of Mavacamten on electromechanical dispersion and deformation in obstructive hypertrophic cardiomyopathy. Circ Heart Fail. 2024;17:e011188. 38502728 10.1161/CIRCHEARTFAILURE.123.011188

[41] Badr A, Roehl K, Suppah M, Abo Abdullah H, Arsanjani R, Siontis KC, et al. Temporal patterns of Holter-detected arrhythmias in hypertrophic cardiomyopathy patients treated with Mavacamten. Biomedicines. 2025;13:1005. 40299669 10.3390/biomedicines13041005 PMC12025015

[42] Castrichini M, Alsidawi S, Geske JB, Newman DB, Arruda-Olson AM, Bos JM, et al. Incidence of newly recognized atrial fibrillation in patients with obstructive hypertrophic cardiomyopathy treated with Mavacamten. Heart Rhythm. 2024;21:2065–7. 38621499 10.1016/j.hrthm.2024.04.055

[43] Kitaoka H, Ieda M, Ebato M, et al. Phase 3 open-label study evaluating the efficacy and safety of Mavacamten in Japanese adults with obstructive hypertrophic cardiomyopathy - the HORIZON-HCM study. Circ J. 2024;89:130–8. 39505542 10.1253/circj.CJ-24-0501

[44] Desai MY, Hajj-Ali A, Rutkowski K, Ospina S, Gaballa A, Emery M, et al. Real-world experience with Mavacamten in obstructive hypertrophic cardiomyopathy: observations from a tertiary care center. Prog Cardiovasc Dis. 2024;86:62–8. 38354765 10.1016/j.pcad.2024.02.001

[45] Boyle TA, Reza N, Hyman M, et al. Atrial fibrillation in patients receiving Mavacamten for obstructive hypertrophic cardiomyopathy: Real-World Incidence, Management, and outcomes. JACC Clin Electrophysiol. 2025;11:411–3. 39641697 10.1016/j.jacep.2024.10.014 PMC12049075

[46] Yildiz M, Muuse J, Schmidt C, Palmer C, Pelchovitz DJ, Schloss EJ, et al. Mavacamten and atrial fibrillation risk: insights from a single-center prospective study. J Am Coll Cardiol. 2025;85:1531–1531.

[47] Saberi S, Cardim N, Yamani M, et al. Mavacamten favorably impacts cardiac structure in obstructive hypertrophic cardiomyopathy: EXPLORER-HCM cardiac magnetic resonance substudy analysis. Circulation. 2021;143:606–8. 33190524 10.1161/CIRCULATIONAHA.120.052359

[48] Desai MY, Okushi Y, Gaballa A, et al. Serial changes in ventricular strain in symptomatic obstructive hypertrophic cardiomyopathy treated with mavacamten: insights from the VALOR-HCM trial. Circ Cardiovasc Imaging. 2024;17:e017185. 39221824 10.1161/CIRCIMAGING.124.017185 PMC11410149

[49] Desai MY, Okushi Y, Wolski K, et al. Mavacamten-Associated Temporal changes in left atrial function in obstructive HCM: insights from the VALOR-HCM trial. JACC Cardiovasc Imaging. 2025;18:251–62. 39254622 10.1016/j.jcmg.2024.08.005

[50] Hegde SM, Lester SJ, Solomon SD, et al. Effect of mavacamten on echocardiographic features in symptomatic patients with obstructive hypertrophic cardiomyopathy. J Am Coll Cardiol. 2021;78:2518–32. 34915982 10.1016/j.jacc.2021.09.1381

[51] Tian Z, Li X, Li L, et al. Effect of Mavacamten on echocardiographic features in Chinese patients with obstructive hypertrophic cardiomyopathy: results from the EXPLORER-CN study. Cardiol Ther. 2025;14:267–82. 40299193 10.1007/s40119-025-00409-5 PMC12084482

[52] Cresci S, Bach RG, Owens AT, Lakdawala NK, Saberi S, Hegde SM, Nilles EK, Wojdyla DM, Sehnert AJ, Wang A. (2025) Response to Mavacamten in patients with high baseline left ventricular filling pressures in the EXPLORER-HCM trial. Circ Cardiovasc Imaging e017824. 10.1161/CIRCIMAGING.124.017824 PMC12684816 40197134

[53] Cremer PC, Geske JB, Owens A, et al. Myosin inhibition and left ventricular diastolic function in patients with obstructive hypertrophic cardiomyopathy referred for septal reduction therapy: insights from the VALOR-HCM study. Circ Cardiovasc Imaging. 2022;15:e014986. 36335645 10.1161/CIRCIMAGING.122.014986

[54] Spertus JA, Fine JT, Elliott P, et al. Mavacamten for treatment of symptomatic obstructive hypertrophic cardiomyopathy (EXPLORER-HCM): health status analysis of a randomised, double-blind, placebo-controlled, phase 3 trial. Lancet. 2021;397:2467–75. 34004177 10.1016/S0140-6736(21)00763-7

[55] Wheeler MT, Olivotto I, Elliott PM, et al. Effects of mavacamten on measures of cardiopulmonary exercise testing beyond peak oxygen consumption: a secondary analysis of the EXPLORER-HCM randomized trial. JAMA Cardiol. 2023;8:240–7. 36652223 10.1001/jamacardio.2022.5099 PMC9857843

[56] Arbelo E, Protonotarios A, Gimeno JR, et al. 2023 ESC guidelines for the management of cardiomyopathies: developed by the task force on the management of cardiomyopathies of the European society of cardiology (ESC). Eur Heart J. 2023;44:3503–626. 37622657 10.1093/eurheartj/ehad194

[57] Ommen SR, Ho CY, Asif IM, et al. 2024 AHA/ACC/AMSSM/HRS/PACES/SCMR guideline for the management of hypertrophic cardiomyopathy: A report of the American heart Association/American college of cardiology joint committee on clinical practice guidelines. Circulation. 2024;149:e1239–311. 38718139 10.1161/CIR.0000000000001250

[58] Aimo A, Todiere G, Barison A, Tomasoni D, Panichella G, Masri A, et al. Diagnosis and management of hypertrophic cardiomyopathy: European vs. American guidelines. Heart Fail Rev. 2025;30:315–25. 39520615 10.1007/s10741-024-10464-0

[59] Amr A, Kayvanpour E, Reich C, Koelemen J, Asokan S, Frey N, et al. Assessing the applicability of cardiac myosin inhibitors for hypertrophic cardiomyopathy management in a large single center cohort. Rev Cardiovasc Med. 2024;25:225. 39076310 10.31083/j.rcm2506225 PMC11270100

[60] Bertero E, Chiti C, Schiavo MA, et al. Real-world candidacy to Mavacamten in a contemporary hypertrophic obstructive cardiomyopathy population. Eur J Heart Fail. 2024;26:59–64. 38131253 10.1002/ejhf.3120

[61] de Gregorio C, Bellocchi P, Napoli AR, et al. Myosin-ATPase inhibitor in real-world patients with obstructive HCM: a report by the cardiomyopathies and pericardial diseases WG of the Italian society of cardiology. J Cardiovasc Med (Hagerstown). 2025;26:381–5. 40575860 10.2459/JCM.0000000000001746

[62] Parodi A, Puscas T, Réant P, et al. Target population for a selective cardiac myosin inhibitor in hypertrophic obstructive cardiomyopathy: real-life estimation from the French register of hypertrophic cardiomyopathy (REMY). Arch Cardiovasc Dis. 2024;117:427–32. 38762345 10.1016/j.acvd.2024.04.001

[63] Abdelfattah OM, Lander B, Demarco K, Richards K, Dubose D, Martinez MW. Mavacamten short-term hemodynamic, functional, and electrocardiographic outcomes: initial real-world post-trial experience. JACC Adv. 2023;2:100710. 38938484 10.1016/j.jacadv.2023.100710 PMC11198066

[64] Becker F, Novotny J, Jansen N, Clauß S, Möller-Dyrna F, Specht B, Orban M, Massberg S, Kääb S, Reichart D. Real-world experience in initiation of treatment with the selective cardiomyosin inhibitor Mavacamten in an outpatient clinic cohort during the 12-week Titration period. Clin Res Cardiol. 2024. 10.1007/s00392-024-02544-w. 39377920 10.1007/s00392-024-02544-w PMC13083493

[65] Kim DS, Chu EL, Keamy-Minor EE, et al. One-year real-world experience with Mavacamten and its physiologic effects on obstructive hypertrophic cardiomyopathy. Front Cardiovasc Med. 2024;11:1429230. 39314763 10.3389/fcvm.2024.1429230 PMC11417615

[66] Ramonfaur D, Gasperetti A, Blake VE, Rivers B, Kassamali AA, Kasper EK, et al. Eighteen-month real-world experience using Mavacamten for treatment of obstructive hypertrophic cardiomyopathy in a racially diverse population. J Am Heart Assoc. 2024;13:e034069. 39082420 10.1161/JAHA.123.034069 PMC11964038

[67] Reza N, Dubey A, Carattini T, Marzolf A, Hornsby N, de Feria A, et al. Real-world experience and 36-week outcomes of patients with symptomatic obstructive hypertrophic cardiomyopathy treated with Mavacamten. JACC: Heart Failure. 2024;12:1123–5. 38661589 10.1016/j.jchf.2024.03.009 PMC11156526

[68] Abood Z, Jan MF, Ashraf M, et al. Real-world assessment of mavacamten’s impact on left ventricular systolic and diastolic functions in obstructive hypertrophic cardiomyopathy: a 1-year single-center observational study. Am J Cardiol. 2025;242:68–74. 39894330 10.1016/j.amjcard.2025.01.032

[69] Burford E, Kasolo Y, Draper J, Sheikh N, Carr-White G, de Marvao A, et al. Tale of three services: early UK experience with Mavacamten treatment for hypertrophic cardiomyopathy with left ventricular outflow tract obstruction. Open Heart. 2025;12:e003218. 40246577 10.1136/openhrt-2025-003218 PMC12007023

[70] Lim J, Cho JY, Kwak S, et al. Real-world experience of Mavacamten for patients with obstructive hypertrophic cardiomyopathy in South korea: a prospective multi-center observational study. Korean Circ J. 2025. 10.4070/kcj.2024.0443. 40169351 10.4070/kcj.2024.0443 PMC12046305

[71] Martinez MW, Seto D, Cheung M, Coiro M, Patel N, Bastien A, Lockman J, Afsari S, Desai MY. Mavacamten: initial insights from the risk evaluation and mitigation strategy program. JACC Adv. 2025;4:101430. 39735613 10.1016/j.jacadv.2024.101430 PMC11681857

[72] Yukselen Z, Raju AKV, Kumar PA, Ujjawal A, Dasari M, Parajuli S, et al. A real–world pharmacovigilance study of FDA adverse event reporting system (FAERS) for Mavacamten. Am J Cardiovasc Drugs. 2024;24:791–9. 39164512 10.1007/s40256-024-00672-2

[73] McGurk KA, Bilgehan N, Ware JS. Pharmacogenetic influences over Mavacamten pharmacokinetics: considerations for the treatment of individuals with hypertrophic cardiomyopathy. Circulation. 2024;149:1786–8. 38829931 10.1161/CIRCULATIONAHA.123.066916 PMC7616064

[74] Perera V, Gretler DD, Seroogy JD, Chiang M, Palmisano M, Florea V. Effects of omeprazole and verapamil on the pharmacokinetics, safety, and tolerability of mavacamten: two drug-drug interaction studies in healthy participants. Clin Pharmacol Drug Dev. 2023;12:1241–51. 37771180 10.1002/cpdd.1332

[75] Rao Gajula SN, Talari S, Nathani TN, Munjal V, Rahman Z, Dandekar MP, et al. Effect of chronopharmacology and food on in vivo pharmacokinetic profile of Mavacamten. Bioanalysis. 2023;15:695–706. 37254776 10.4155/bio-2023-0030

[76] Cotté F-E, Hurst M, Akarkoub S, Ho M, Vernon J, Chambry L, Leproust S. Cost-effectiveness of Mavacamten for the treatment of patients with symptomatic obstructive hypertrophic cardiomyopathy using a French healthcare perspective. Eur J Health Econ. 2025. 10.1007/s10198-025-01808-0. 40549096 10.1007/s10198-025-01808-0 PMC12929265

[77] Desai N, Xie J, Wang Y, Sutton MB, Whang J, Fine JT, Garrison LP. Projecting the Long-term clinical value of Mavacamten for the treatment of obstructive hypertrophic cardiomyopathy in the united states: an assessment of net health benefit. Clin Ther. 2022;44:52–e662. 34911641 10.1016/j.clinthera.2021.11.006

[78] Wheeler MT, Jacoby D, Elliott PM, et al. Effect of beta-blocker therapy on the response to Mavacamten in patients with symptomatic obstructive hypertrophic cardiomyopathy. Eur J Heart Fail. 2023;25:260–70. 36404399 10.1002/ejhf.2737

[79] Dominguez F, Cabrera E. Mavacamten in obstructive hypertrophic cardiomyopathy – are beta-blockers blocking part of its shine? Eur J Heart Fail. 2023;25:271–3. 36597820 10.1002/ejhf.2768

[80] Sherrid MV, Barac I, McKenna WJ, Elliott PM, Dickie S, Chojnowska L, et al. Multicenter study of the efficacy and safety of disopyramide in obstructive hypertrophic cardiomyopathy. J Am Coll Cardiol. 2005;45:1251–8. 15837258 10.1016/j.jacc.2005.01.012

[81] Willeford A, Silva Enciso J. Transitioning disopyramide to Mavacamten in obstructive hypertrophic cardiomyopathy: a case series and clinical guide. Pharmacotherapy. 2023;43:1397–404. 37688422 10.1002/phar.2874

[82] Maron MS, Masri A, Nassif ME, et al. Impact of Aficamten on disease and symptom burden in obstructive hypertrophic cardiomyopathy: results from SEQUOIA-HCM. J Am Coll Cardiol. 2024;84:1821–31. 39352339 10.1016/j.jacc.2024.09.003

[83] Garcia-Pavia P, Bilen O, Burroughs M, et al. Aficamten vs Metoprolol for obstructive hypertrophic cardiomyopathy: MAPLE-HCM Rationale, study Design, and baseline characteristics. JACC Heart Fail. 2025;13:346–57. 39909646 10.1016/j.jchf.2024.11.011

[84] Shah SJ, Rigolli M, Javidialsaadi A, et al. Cardiac myosin inhibition in heart failure with normal and supranormal ejection fraction: primary results of the EMBARK-HFpEF trial. JAMA Cardiol. 2025;10:170–5. 39347697 10.1001/jamacardio.2024.3810 PMC11822545

[85] Almeida-Coelho J, Leite-Moreira AM, Sequeira V, Hamdani N, Lourenço AP, Falcão-Pires I, et al. Myosin-inhibitor mavacamten acutely enhances cardiomyocyte diastolic compliance in heart failure with preserved ejection fraction. Circ Heart Fail. 2024;17:e011833. 39239713 10.1161/CIRCHEARTFAILURE.124.011833

[86] Desai MY, Owens AT, Abraham T, et al. Mavacamten in symptomatic nonobstructive hypertrophic cardiomyopathy. N Engl J Med. 2025. 10.1056/NEJMoa2505927. 40888717 10.1056/NEJMoa2505927

[87] Dufton C, Evanchik M, Daniel DD, Silverman JA, Marilyn MM, Madden M, et al. EDG-7500, a first-in-class cardiac sarcomere modulator, demonstrates favorable tolerability, safety, and pharmacokinetics in healthy adults. J Card Fail. 2025;31:345.

[88] Saleh D, Kim EY, Hussain K, et al. Anterior mitral valve leaflet length and response to Mavacamten in obstructive hypertrophic cardiomyopathy. Eur Heart J. 2025;3:qyaf081. 10.1093/ehjimp/qyaf081 PMC12214459 40606967

